# First-in-human clinical study of novel technique to diagnose malignant melanoma via thermal conductivity measurements

**DOI:** 10.1038/s41598-019-40444-6

**Published:** 2019-03-07

**Authors:** Takahiro Okabe, Taku Fujimura, Junnosuke Okajima, Yumi Kambayashi, Setsuya Aiba, Shigenao Maruyama

**Affiliations:** 1 Graduate School of Science and Technology, Hirosaki University, Hirosaki, Japan; 2 Graduate School of Medicine, Tohoku University, Sendai, Japan; 3 Institute of Fluid Science, Tohoku University, Sendai, Japan; 4 National Institute of Technology, Hachinohe College, Hachinohe, Japan

## Abstract

Melanoma is an aggressive skin cancer that originates from melanocytes and, especially in the case of early-stage melanoma, is distributed adjacent to the epidermis and superficial dermis. Although early-stage melanoma can be distinguished from benign nevus via a dermoscopy, it is difficult to distinguish invasive melanoma in its early stages from *in situ* melanoma. Because invasive melanoma must undergo a sentinel lymph node biopsy to be diagnosed, a non-invasive method to detect the micro-invasion of early-stage melanoma is needed for dermato-oncologists. This paper proposes a novel quantitative melanoma identification method based on accurate measurements of thermal conductivity using a pen-shaped device. This method requires skin temperature data for one minute to determine the effective thermal conductivity of the skin, allowing it to distinguish melanoma lesions from healthy skin. Results suggest that effective thermal conductivity was negative for *in situ* melanoma. However, in accordance with tumour progression, effective thermal conductivity was larger in invasive melanoma. The proposed thermal conductivity measurement is a novel tool that detects the micro-invasion of melanoma.

## Introduction

Melanoma is an aggressive skin cancer that originates from melanocytes and exhibits a malignant transformation distributed throughout the epidermis and superficial dermis, as it can spread through blood and lymphatic vessels^1^. Therefore, it is important to distinguish *in situ* melanoma from dermal invasive melanoma. Dermoscopy is a non-invasive, widely used technique that has had a significant impact on the early diagnosis of invasive and *in situ* melanoma^2,3^. Generally, in clinics, after diagnosing melanoma by using a dermoscope, dermatologists perform a skin biopsy prior to the radical resection of the tumour to evaluate tumour thickness, the final determination of which takes approximately one week^2–5^. In addition, the accuracy of the first diagnosis using only dermoscopy strongly depends on the dermatologist’s skill^6^. While dermoscopy is a useful tool to distinguish *in situ* melanoma from nevus pigmentosus, it is not as useful in distinguishing *in situ* melanoma from micro-invasive melanoma. Therefore, there is a strong need for an objective and quantitative diagnosis instrument that could immediately distinguish *in situ* melanoma from invasive melanoma. Although many researchers have proposed quantitative diagnosis methods such as laser-based systems and IR imaging systems, these have still not been transferred into practical use, because of how long they take and their lack of portability. Furthermore, any method which relies on optical or imaging systems still requires complex and expensive equipment.

Here, we propose a novel quantitative identification method for melanoma based on accurate measurements of thermal conductivity. This method uses a guard-heated thermistor probe, which is a pen-shaped device that can accurately and rapidly measure the absolute value of skin surface temperature and the effective thermal conductivity of the skin^7,8^. The main advantages of this method are its high sensitivity, short turnaround time, ease of use, and portability. This method only requires skin temperature data for one minute to determine the effective thermal conductivity of the skin. It can distinguish melanoma types by measuring differences in effective thermal conductivity between lesions and healthy skin. The feasibility of our method is tested with eleven melanoma patients including those with early-stage melanoma. The thermal conductivity measurement can thus be a novel option for melanoma diagnosis.

## Results

### Comparison between effective thermal conductivities of lesions and healthy skin on ***in situ*** melanoma patients

**Figure 1:**
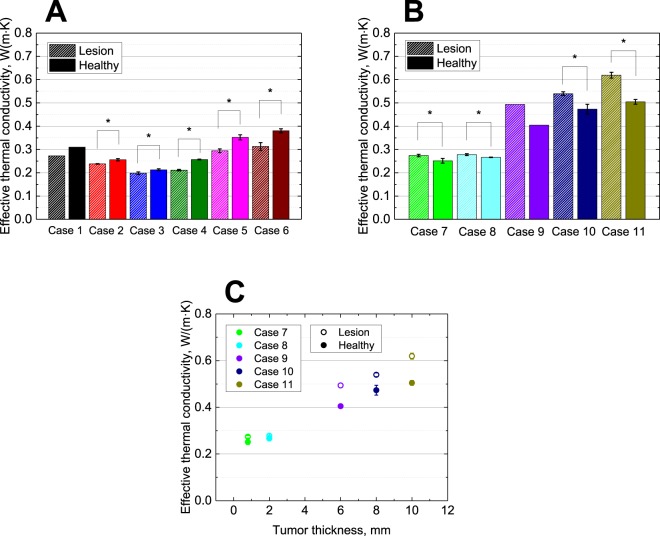
(**A**) Comparison of effective thermal conductivities (W/(m.K)) between healthy skin and lesions measured *in situ* for melanoma patients (Cases 1–6). Data are presented as mean ± SD (*n* = 3). (**B**) Comparison of effective thermal conductivities between healthy skin and lesions measured for invasive melanoma patients (Cases 7–11). Data are presented as mean ± SD (*n* = 3). (**C**) Correlation between effective thermal conductivity and tumour thickness (mm) for invasive melanoma patients (Cases 7–11). Statistical significance is indicated by *P < 0.05 (Mann–Whitney U-test).

Results of effective thermal conductivities measured on *in situ* melanoma patients (Cases 1–6) are detailed in Table 1 and Fig. 1A. The *in situ* melanoma group exhibited significantly lower values of effective thermal conductivity at lesions when compared to healthy skin (*P* = 0.0495, *k*_l_ *vs. k*_h_ in Cases 2–6). Effective thermal conductivities varied within the 0.198–0.380 W/(m∙K) range, depending on body locations or age. The maximum difference between lesions and healthy skin was observed in Case 6, while the minimum was observed in Case 3. Tumour size was not correlated with the effective thermal conductivity.

**Table 1:** Mean values of effective thermal conductivity and skin surface temperature measured for lesion and healthy skin.

	Lesion		Healthy skin		*k*_l_ − *k*_h_ [W/(m·K)]	*P-value*
	*k*_l_ [W/(m·K)]	*T* [°C]	*k*_h_ [W/(m·K)]	*T* [°C]		
Case 1 (*n* = 1)	0.272	29.67	0.310	29.82	−0.018	—
Case 2	0.238 ± 0.002	27.30 ± 0.09	0.256 ± 0.005	27.61 ± 0.03	−0.018	0.0495
Case 3	0.198 ± 0.006	30.12 ± 0.13	0.213 ± 0.004	28.86 ± 0.06	−0.015	0.0495
Case 4	0.211 ± 0.003	30.33 ± 0.03	0.257 ± 0.002	30.56 ± 0.15	−0.046	0.0495
Case 5	0.295 ± 0.008	33.88 ± 0.17	0.352 ± 0.011	33.72 ± 0.19	−0.058	0.0495
Case 6	0.313 ± 0.017	32.52 ± 0.09	0.380 ± 0.009	32.44 ± 0.17	−0.067	0.0495
Case 7	0.274 ± 0.005	34.29 ± 0.50	0.251 ± 0.010	34.42 ± 0.26	0.023	0.0495
Case 8	0.278 ± 0.004	31.65 ± 0.12	0.266 ± 0.001	32.04 ± 0.05	0.012	0.0495
Case 9 (*n* = 1)	0.493	31.87	0.405	31.21	0.089	—
Case 10	0.539 ± 0.008	31.88 ± 0.19	0.473 ± 0.002	32.46 ± 0.37	0.066	0.0495
Case 11	0.619 ± 0.012	35.72 ± 0.29	0.505 ± 0.010	36.33 ± 0.07	0.115	0.0495

Next, we investigated the relationship of the effective thermal conductivities to skin surface temperatures measured immediately before the pulse heating. We observed a positive correlation between effective thermal conductivity and skin surface temperature. Skin surface temperatures varied widely from 27–34 °C, depending on body locations (Table 1 and Fig. S1A). At the plantar and heel, surface temperatures were relatively low compared to those at the lower thigh and waist. Furthermore, effective thermal conductivities were also smaller at the plantar and heel. These results were consistent with those of our previous study^7^.

### Comparison between effective thermal conductivities of lesion and healthy skin on invasive melanoma patients

Results of effective thermal conductivities measured on invasive melanoma patients (Cases 7–11) are shown in Table 1 and Fig. 1B. Effective thermal conductivities varied from 0.251–0.612 W/(m∙K), which were larger than those in the *in situ* melanoma group. The effective thermal conductivities at lesions were significantly higher than those at healthy skin (*P* = 0.0495, *k*_l_ *vs. k*_h_ in Cases 7, 8, 10, 11), which is the opposite of the *in situ* melanoma group. A significantly higher effective thermal conductivity associated with the progression of tumour growth was observed in Cases 9–11. The invasive melanoma group exhibited relatively high skin surface temperatures compared to the *in situ* melanoma group, due to increased blood perfusion and metabolic activity from tumour growth (Table 1 and Fig. S1B). This result was consistent with those of previous studies^9,10^. We found a significant positive correlation between effective thermal conductivity and tumour thickness (Fig. 1C). Differences compared to healthy skin also increased with the progression of tumour growth.

### Difference in temperature responses between lesions and healthy skin

**Figure 2:**
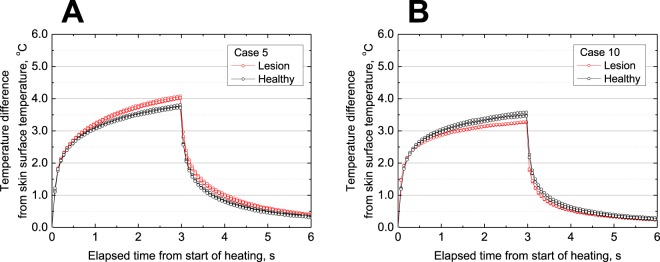
Raw thermal conductivity measurements on (**A**) *in situ* melanoma and (**B**) invasive melanoma. Red and black lines show lesion and healthy skin data, respectively. Results of three measurements are plotted for each data type.

Figure 2 details temperature responses during and after pulse heating between lesions and healthy skin. Figure 2A,B show typical temperature responses obtained from the measurements on *in situ* (Case 5) and invasive (Case 10) melanoma patients, respectively. The differences in temperature responses between lesions and healthy skin were 0.1–0.6 °C, which is detectable by the guard-heated thermistor probe that has a temperature resolution of 0.005 °C. The repeatability of the measurements was sufficient for detecting differences.

## Discussion

Although dermoscopy is useful to distinguish atypical nevus from *in situ* melanoma^3^, it is difficult to distinguish micro-invasive melanoma from *in situ* melanoma^2,3^. Because it is difficult in some cases to distinguish micro-invasive melanoma from *in situ* melanoma using only dermoscopy^3^, and it takes approximately one week to make a histological diagnosis using the skin biopsy results, a novel device that can detect dermal micro-invasion of melanoma is needed during early stages to avoid unnecessary surgery for melanoma patients^1,5^.

Here we investigated invasive and non-invasive melanoma patients to detect the threshold of dermal invasion using thermal conductivity measurements of the skin surface. We have previously developed a novel device that can detect absolute surface temperature with a temperature resolution 100 times higher than that of conventional sensors such as thermocouple and IR thermography. This device can also be used for a non-invasive and accurate measurement of the effective thermal conductivity of human skin by measuring skin surface temperature response for a short time.

Because skin surface temperature, which is regulated by the local metabolism, and blood perfusion underneath the skin, is a reflection of the physiological state of the human body^11^, many studies have attempted to apply skin surface temperature information to melanoma diagnostic techniques. Most medical diagnosis studies involving skin surface temperature have used infrared (IR) thermography. These methods have great advantages such as a non-invasive modality and a large amount of data, meaning a temperature distribution can be attained over the entire visible surface. They can be generally separated into two types: a passive method that only measures skin surface temperature^9,10^, and an active method that measures the temperature response on the skin surface when heating or cooling is applied to enhance or induce thermal contrast^12–14^. Results of recent studies based on IR imaging suggested that the passive method can only detect larger progressive lesions with significant temperature increases, compared to the surrounding normal skin^9,10^. The active method allows for the detection of relatively small melanoma lesions such as a 2 mm diameter, 0.44 mm deep lesion^12–14^. However, it was difficult to detect a significant temperature difference for an *in situ* melanoma lesion even using the active method. Thus, smaller lesions that cause smaller temperature differences require a more accurate, detection method.

Compared to IR imaging approaches, our method offers advantages in the detection of *in situ* melanoma lesions, for which high sensitivity and accuracy are essential. We successfully detected a negative effective thermal conductivity in *in situ* melanoma compared to the surrounding healthy skin. These results show a possibility that the thermal conductivity of tumour cells is lower than that of keratinocytes before the tumour growth progression. There were no false positives or false negatives detected using the guard-heated thermistor probe.

In contrast, effective thermal conductivity increased in invasive melanoma, in accordance with tumour growth. This is because the tumour cell invasion reached the dermal layer, leading to a local increase in blood and lymph flows around the tumour, through the formation of cancer stroma. Melanomas induce the growth of new capillary blood vessels by producing specific angiogenesis-promoting growth factors. New blood-vessel growth continues through the progression from precancerous skin lesions to full-blown skin cancer. The presence of new blood vessels and the increased blood supply affect the temperature response^15^, resulting in an increase in the effective thermal conductivity of lesions. The increase in effective thermal conductivity depends on the melanoma stage. These results support the idea that our method can be applied to distinguish invasive melanoma in its early stages from *in situ* melanoma.

The other advantages of our method are shorter required measurement time, smaller temperature changes, and ease of use. Our method only requires skin temperature data for one minute to determine the effective thermal conductivity of the skin, leading to its ability to distinguish melanoma lesions from healthy skin. The required heating induces only a tiny increase in skin temperature; therefore, the duration of measurement and temperature rise never causes significant discomfort to the patient.

In conclusion, our study identified a novel high-accuracy diagnostic technique for malignant melanoma. We revealed that effective thermal conductivity varies depending on the stage of lesions. Therefore, our method has the potential to differentiate between early-stage melanoma and invasive melanoma. Although we investigated the effective thermal conductivity as a diagnostic tool for skin cancer, the exact pathological mechanisms of the correlation between the effective thermal conductivity and stromal factors of melanoma are still unclear. These might be explained by considering stromal factors such as extracellular matrix proteins. To confirm this hypothesis, further immunohistochemical pathological studies are required. Because this was a pilot study, future independent studies with larger patient groups are required to confirm our finding.

## Patients and Method

### Study oversight

The study protocol and all amendments were approved by the institutional review board at Tohoku University Graduate School of Medicine (2016-2-285). The study was conducted in accordance with the Declaration of Helsinki and with Good Clinical Practice guidelines as defined by the International Conference on Harmonisation. All patients provided written informed consent before enrolment. A data and safety monitoring committee was established to provide oversight of safety and efficacy considerations. The study was registered with UMIN (UMIN000025618).

### Study design

Patient characteristics are listed in Table 2. Eleven patients were treated at Tohoku University Hospital, Sendai, Japan from March–December 2017. All patients had the effective thermal conductivity of their skin measured prior to the conventional treatment (Table 1). Six of the treated subjects were histologically diagnosed as *in situ* melanoma (stage 0), and the other five subjects were diagnosed as invasive melanoma (stages I–IV). The diagnosis of melanoma was made independently by two dermato-pathologists and a pathologist at Tohoku University.

**Table 2:** Patient characteristics.

	Sex	Age (years)	Locations	Stage	Epidermis thickness, mm	Tumour size, mm × mm	Tumour thickness, mm
Case 1	F	60	planter	pTisN0M0 stage 0	0.83	10 × 10	—
Case 2	F	84	heel	pTisN0M0 stage 0	1.4	30 × 28	—
Case 3	M	71	plantar	pTisN0M0 stage 0	0.20	15 × 10	—
Case 4	M	70	lower thigh	pTisN0M0 stage 0	0.10	40 × 40	—
Case 5	M	65	waist	pTisN0M0 stage 0	0.10	19 × 15	—
Case 6	F	21	lower thigh	pTisN0M0 stage 0	0.20	15 × 10	—
Case 7	M	85	finger	pT1aN0M0 stage I A	0.20	7 × 7	0.8
Case 8	F	84	plantar	pT2aN0M0 stage II B	0.28	27 × 23	2
Case 9	F	65	plantar	pT4bN0M0 stage II C	0.60	28 × 18	6
Case 10	M	68	femur	pT4aN3cM1 stage IV	0.10	8 × 8	8
Case 11	F	39	lower thigh	pT4aN0M0 stage IV	0.15	10 × 10	10

### Experimental setup

**Figure 3:**
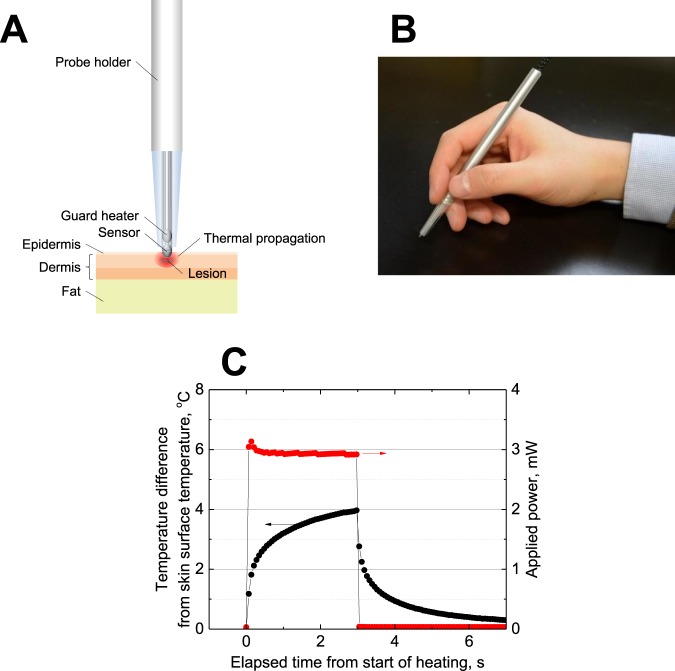
(**A**) Schematic diagram of a guard-heated thermistor probe placed above a lesion. (**B**) Picture of a guard-heated thermistor probe used in clinical experiments. (**C**) The heating protocol of a thermal conductivity measurement. The temperature (°C) response of the thermistor when the pulse power is applied for 3 s is shown. Red and black lines indicate temperature differences from applied power and skin surface temperatures, respectively.

We used a guard-heated thermistor probe, developed in our previous studies^7,8^, to accurately and rapidly measure the effective thermal conductivity of the skin. Figure 3A,B show images of a guard-heated thermistor probe and a pen-shaped probe for clinical use, respectively. The probe consists of two glass-coated thermistors (PSB-S9 type, Shibaura Electronics Co., Ltd) which were previously calibrated using a standard thermometer system (1560 black stack thermometer and 5640 thermistor standard probe, FLUKE), and then inserted into a pen-shaped probe holder for practical use and solidity. One of the thermistors is used as a measuring sensor to contact with the skin and the other is used as a guard heater. As demonstrated in previous work^8^, the guard heater minimizes heat loss along lead wires, which can cause significant errors in temperature readings. The probe has a temperature resolution of ~100 times better than conventional sensors such as thermocouple and IR thermography.

### Principle of thermal conductivity measurement

We employed a pulse-power integrated-decay technique proposed by Kharalkar *et al*.^16^. In this technique, the thermal conductivity is based on a measurement of the thermistor temperature response after pulse power heating for a few seconds (Fig. 3C). This technique offers the advantage of a short-duration measurement and does not require the specific heat and density of a specimen prior to the experiment. Thermal conductivity can be determined as follows:

1 $$k={({a}_{1}\frac{{\int }_{t-{t}_{{\rm{h}}}}^{t-{t}_{{\rm{h}}}+3.0}{\rm{\Delta }}T(t)dt}{{\int }_{0}^{{t}_{{\rm{h}}}}P(t)dt}-{a}_{2})}^{-1},$$

where *k*, *t*, *t*_h_, *P*, and *ΔT* denote thermal conductivity [W/(m∙K)], time [s], heating time [s], power [W], and temperature difference [°C], respectively. *a*_1_ and *a*_2_ are constants determined experimentally for the apparatus. The apparatus constants were previously determined by the measurements on two standard materials (silicone rubber and 1.0 wt% agar-gelled water) with known thermal conductivities similar to that of human skin.

### Experimental procedure

The experimental procedure used was similar to the one in our previous study^7^; thus, we provide a brief explanation here. The effective thermal conductivity of the skin was measured in a room where the temperature and the relative humidity were maintained at 21 ± 1 °C and 50 ± 3%, respectively. During the measurement process, a half-exposed thermistor in the probe tip makes contact with the skin surface and measures the temperature of the skin for 50 s. Subsequently, a pulse power of 3 mW is applied for 3 s to the thermistor. After heating, the temperature decay is measured for an additional 3 s. To minimize heat loss along the lead wires during measurement, heat generation of the thermistor for the guard heater is controlled to minimize the temperature difference between the thermistors. Measurements were performed three times on both the lesion and healthy skin of each volunteer. The healthy skin measurements were performed as close to the lesions as possible to avoid effects from skin structure differences. Afterward, we distinguish melanoma based on the difference in effective thermal conductivity between the healthy skin and lesion.

### Statistical analysis

All measurements are presented as mean ± SD. Differences between lesion and healthy skin were determined using the Mann–Whitney U-test. A *P* value < 0.05 was considered to represent a statistically significant result.

## Supplementary information

Supplementary Information
