# Supplementary material for: First-in-human clinical study of novel technique to diagnose malignant melanoma via thermal conductivity measurements
Source: Sci Rep. 2019 Mar 7;9:3853. doi: 10.1038/s41598-019-40444-6 (PMC6405870; doi:10.1038/s41598-019-40444-6)
Supplement: Supplementary file 1 — Supplementary Information [file 41598_2019_40444_MOESM1_ESM.docx]

**Supplementary Information**

First-in-human clinical study of novel technique to diagnose malignant melanoma via thermal conductivity measurements

Takahiro Okabe^1, *^, Taku Fujimura^2, *^, Junnosuke Okajima^3^, Yumi Kambayashi^2^, Setsuya Aiba^2^, Shigenao Maruyama^4^

^1^Graduate School of Science and Technology, Hirosaki University, Japan.

^2^Graduate School of Medicine, Tohoku University, Japan.

^3^Institute of Fluid Science, Tohoku University, Japan.

^4^National Institute of Technology, Hachinohe College, Japan.

^*^ Equal contribution

**Corresponding author:**

Takahiro Okabe, Ph.D. (Eng.)

Graduate School of Science and Technology

Hirosaki University, 3 Bunkyo-cho, Hirosaki, Aomori, 036-8561, Japan

Tel: +81-172-39-3615

E-mail: oka@hirosaki-u.ac.jp

**Supplementary Figure:**

**A**

**B**

**Figure S1.** (A) Effective thermal conductivity as a function of skin surface temperature (°C) measured in situ for melanoma patients. Skin surface temperatures varied widely from 27 – 34°C, depending on body locations. (B) Effective thermal conductivities as a function of skin surface temperature measured for invasive melanoma patients. The invasive melanoma group exhibited relatively high skin surface temperatures compared to the in situ melanoma group, due to increased blood perfusion and metabolic activity from tumour growth.
